# QSAR Analysis of 2-Amino or 2-Methyl-1-Substituted Benzimidazoles Against *Pseudomonas aeruginosa*

**DOI:** 10.3390/ijms10041670

**Published:** 2009-04-17

**Authors:** Sanja O. Podunavac-Kuzmanović, Dragoljub D. Cvetković, Dijana J. Barna

**Affiliations:** 1 Faculty of Technology, University of Novi Sad, Bulevar cara Lazara 1, 21000 Novi Sad, Serbia; 2 Institute of Public Health, Zmaj Jovina 30, 24000 Subotica, Serbia

**Keywords:** QSAR, benzimidazole derivatives, Pseudomonas aeruginosa, multiple linear regression

## Abstract

A set of benzimidazole derivatives were tested for their inhibitory activities against the Gram-negative bacterium *Pseudomonas aeruginosa* and minimum inhibitory concentrations were determined for all the compounds. Quantitative structure activity relationship (QSAR) analysis was applied to fourteen of the abovementioned derivatives using a combination of various physicochemical, steric, electronic, and structural molecular descriptors. A multiple linear regression (MLR) procedure was used to model the relationships between molecular descriptors and the antibacterial activity of the benzimidazole derivatives. The stepwise regression method was used to derive the most significant models as a calibration model for predicting the inhibitory activity of this class of molecules. The best QSAR models were further validated by a leave one out technique as well as by the calculation of statistical parameters for the established theoretical models. To confirm the predictive power of the models, an external set of molecules was used. High agreement between experimental and predicted inhibitory values, obtained in the validation procedure, indicated the good quality of the derived QSAR models.

## 1. Introduction

The benzimidazoles are a large chemical family used as antimicrobial agents against the wide spectrum of microorganisms [1–9]. Because of its synthetic utility and broad range of pharmacological effects, the benzimidazole nucleus is an important heterocyclic ring, and interest in the chemistry, synthesis and microbiology of this pharmacophore continues to be fuelled by its antifungal [10], antitubercular [11], antioxidant [12,13], and antiallergic [14,15] properties. Other reports have revealed that these molecules are also present in a variety of antiparasitic [16,17] and herbicidal agents [18]. Albendazole, fenbendazole and their sulphoxide derivatives are methylcarbamate benzimidazoles with a broad spectrum anthelmintic activity, widely used in human and veterinary medicine [19]. They are used against several systemic parasitoses, including nematodoses, hidatidosis, teniasis and others [20]. They are also used to treat microsporodial and cryptosporodial infections, which can cause lethal diarrhea in patients treated with immunosuppressive drugs, or infected with HIV [21,22].

Different substituted benzimidazolyl quinolinyl mercaptotriazoles are remarkably effective compounds both with respect to their virus inhibitory activity and their favourable antibacterial activity [23]. In recent years, benzimidazole derivatives have been attracted particular interest due to their antiviral activity against HCV (Hepatitis C virus) [24,25].

Although a variety of benzimidazole derivatives are known, the development of new and convenient strategies to synthesize new biologically active benzimidazoles is of considerable interest. Quantitative structure activity relationship (QSAR) studies are useful tools in the rational search for bioactive molecules. The main success of the QSAR method is the possibility to estimate the characteristics of new chemical compounds without the need to synthesize and test them. This analysis represents an attempt to relate structural descriptors of compounds with their physicochemical properties and biological activities. This is widely used for the prediction of physicochemical properties in the chemical, pharmaceutical, and environmental spheres. This method included data collection, molecular descriptor selection, correlation model development, and finally model evaluation. QSAR studies have predictive ability and simultaneously provide deeper insight into mechanism of drug receptor interactions [26,27].

In view of the above and in continuation of our studies on the inhibitory activities of benzimidazole derivatives, as well as on correlation of molecular properties with activity [4–8,28–35], the objective of this investigation was to study the usefulness of QSAR in the prediction of the antibacterial activity of benzimidazole derivatives against *Pseudomonas aeruginosa*. Multiple linear regression (MLR) models have been developed as a mathematical equation which can relate chemical structure to the inhibitory activity.

## 2. Results and Discussion

In the first step of the present study, different substituted benzimidazoles (Table 1) were evaluated for *in vitro* antibacterial activity against Gram-negative *Pseudomonas aeruginosa*. The inhibitory effects of compounds **1** – **14**, expressed as minimum inhibitory concentration (MIC) values, are summarized in Table 2.

The screening results reveal that all the compounds exhibited appreciable *in vitro* activity against the tested strain. In the second step, we focused our efforts on developing the QSAR models of compounds **1** – **14** as antibacterial agents. A set of benzimidazoles was used for MLR model generation. The reference drugs were not included in model generation as they belong to a different structural series. Inhibitory activity data determined as μg/mL were first transformed to the negative logarithms of molar MICs (log1/*c*_MIC_), (Table 2) which was used as a dependent variable in the QSAR study. Different physicochemical, steric, electronic, and structural molecular descriptors were used as independent variables and were correlated with antibacterial activity.

Developing a QSAR model requires a diverse set of data, and, thereby a large number of descriptors have to be considered. Descriptors are numerical values that encode different structural features of the molecules. Selection of a set of appropriate descriptors from a large number of them requires a method, which is able to discriminate between the parameters. Pearson's correlation matrix has been performed on all descriptors by using NCSS Statistical Software. The analysis of the matrix revealed nine descriptors for the development of MLR model. The values of descriptors selected for MLR model are presented in Table 3. Linear models were then formed by a stepwise addition of terms. A delition process was then employed, whereby each variable in the model was held out in turn and using the remaining parameters models were generated. Each descriptor was chosen as input for the statistical software package and then the stepwise addition method implemented in the software was used for choosing the descriptors contributing to the antibacterial activity of benzimidazole derivatives.

The specifications for the best-selected MLR models are shown in Table 4. The monoparametric model indicated the importance of molar weight (*MW*) in contribution to inhibitory activity (model 1, Table 4). Addition of total energy (*TE*) as an additional parameter to *MW*, significantly increased the correlation coefficient from 0.7910 to 0.8587 (model 2, Table 4). Similarly, the addition of a third parameter also increased the correlation coefficient, but a MLR method only can be used when a relatively small number of molecular descriptors are used (at least five to six times smaller than the total number of compounds). In this case (for fourteen compounds), only two descriptors can be used to develop a good QSAR model in order to avoid a high chance of spurious correlations. In this approach, only the QSAR models 1 and 2 can be considered.

It is well known that there are three important components in any QSAR study: development of models, validation of models and utility of developed models. Validation is a crucial aspect of any QSAR analysis [36]. The statistical quality of the resulting models, as depicted in Table 4, is determined by *r*, *s*, and *F* [37–39]. It is noteworthy that all these equations were derived using the entire data set of compounds (*n =* 14) and no outliers were identified. The *F*-value presented in Table 4 is found statistically significant at 99% level since all the calculated *F* values are higher as compared to tabulated values.

For the testing the validity of the predictive power of selected MLR models the LOO technique was used. The developed models were validated by the calculation of following statistical parameters: PRESS, SSY, S_PRESS_*, r*^2^ _CV_, and *r*^2^ _adj_ (Table 5). These parameters were calculated from the following equations:

(1) $$\text{PRESS}=\sum {\left(Y_{obs}-Y_{calc}\right)}^{2}$$

(2) $$\text{SSY}=\sum {\left(Y_{obs}-Y_{mean}\right)}^{2}$$

(3) $${\text{S}}_{\text{PRESS}}=\sqrt{\frac{\text{PRESS}}{n}}$$

(4) $$r_{\text{CV}}^{2}=1-\frac{\text{PRESS}}{\text{SSY}}$$

(5) $$r_{\text{adj}}^{2}=1-(r^{2})\left(\frac{n-1}{n-p-1}\right)$$

where, *Y*_obs_, *Y*_calc_ and *Y*_mean_ are observed, calculated and mean values; *n*, number of compounds; *p*, number of independent parameters.

PRESS is an acronym for prediction sum of squares. It is used to validate a regression model with regards to predictability. To calculate PRESS, each observation is individually omitted. The remaining n – 1 observations are used to calculate a regression and estimate the value of the omitted observation. This is done n times, once for each observation. The difference between the actual Y value, y_obs_, and the predicted Y, y_calc_, is called the prediction error. The sum of the squared prediction errors is the PRESS value. The smaller PRESS is, the better the predictability of the model. Its value being less than SSY points out that the model predicts better than chance and can be considered statistically significant. SSY are the sums of squares associated with the corresponding sources of variation. These values are in terms of the dependent variable, y.

The PRESS value above can be used to compute an *r*^2^ _CV_ statistic, called *r*^2^ cross validated, which reflects the prediction ability of the model. This is a good way to validate the prediction of a regression model without selecting another sample or splitting your data. It is very possible to have a high *r*^2^ and a very low *r*^2^ _CV_. When this occurs, it implies that the fitted model is data dependent. This *r*^2^ _CV_ ranges from below zero to above one. When outside the range of zero to one, it is truncated to stay within this range. Adjusted *r*-squared (*r*^2^ _adj_) is an adjusted version of *r*^2^. The adjustment seeks to remove the distortion due to a small sample size.

In many cases *r*^2^ _CV_ and *r*^2^ _adj_ is taken as a proof of the high predictive ability of QSAR models. A high value of these statistical characteristic (> 0.5) is considered as a proof of the high predictive ability of the model, although recent reports have proven the opposite [40]. Although a low value of *r*^2^ _CV_ for the training set can indeed serve as an indicator of a low predictive ability of a model, the opposite is not necessarily true. Indeed, the high *r*^2^ _CV_ does not imply automatically a high predictive ability of the model. Thus, the high value of LOO *r*^2^ _CV_ is the *necessary* condition for a model to have a high predictive power, it is not a *sufficient* condition. It is proven that the only way to estimate the true predictive power of a model is to test it on a sufficiently large collection of compounds from an external test set. The test set must include no less than five compounds, whose activities and structures must cover the range of activities and structures of compounds from the training set. This application is necessary for obtaining trustful statistics for comparison between the observed and predicted activities for these compounds. Besides high *r*^2^ _CV_, a reliable model should be also characterized by a high correlation coefficient between the predicted and observed activities of compounds from a test set of molecules that was not used to develop the models.

To confirm the predictive power of the QSAR models, an external set of benzimidazoles was used. Five benzimidazole derivatives which were tested in our previous paper for their antibacterial activity against the *Pseudomonas aeruginosa* were used as the external set of molecules [33]. In the present paper, the inhibitory activity of the following compounds was calculated: 1-(3-methoxybenzyl)-5,6- dimethylbenzimidazole (**15**), 1-(3-methylbenzyl)-2-aminobenzimidazole (**16**), 1-(3-chlorobenzyl)-2- amino-benzimidazole (**17**), 1-(3-fluorobenzyl)-2-amino-5,6-dimethylbenzimidazole (**18**) and 1-(3- methoxybenzyl)-2-amino-5,6-dimethylbenzimidazole (**19**).

The values of inhibitory activitiy of a test set of molecules was calculated with the models 1 and 2. These data are compared with experimentally obtained values of antibacterial activity against the same bacteria. From the data presented in Table 6, it is shown that high agreement between experimental and predicted inhibitory values was obtained (the residual values are small, indicating the good predictability of the established models. According to the reference [40], without the validation of the QSAR models by using the external test set, we could not have come to a right conclusion about high predictive ability of derived models.

**Figure 1.**
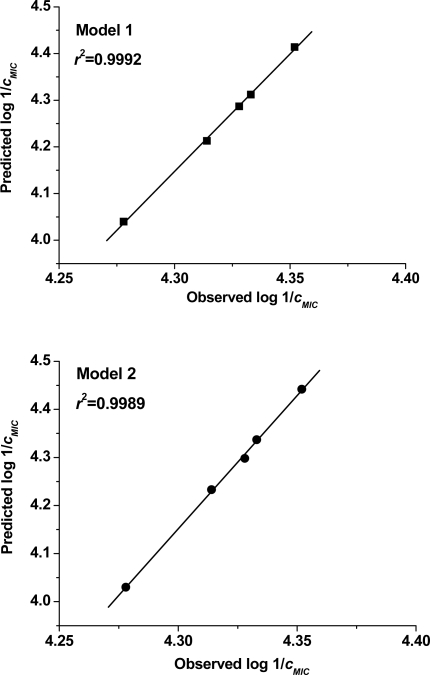
Plots of predicted versus experimentally observed inhibitory activity of benzimidazoles against *Pseudomonas aeruginosa.*

Figure 1 shows the plots of linear regression predicted *versus* experimental values of the antibacterial activity of external set of benzimidazoles. The plots for QSAR models 1 and 2 show a very good fit with *r*^2^ = 0.9992 and 0.9989, respectively. It indicates that models 1 and 2 can be successfully applied to predict the antibacterial activity of these class of molecules. Moreover, it is not possible to use the reported QSAR models to predict the activity of any type of molecules *vs. Pseudomonas aeruginosa.* The applicability domain of the derived QSAR models is the different substituted 1-benzyl or 1-benzoylbenzimidazole derivatives. However, it is very important to point out an eventual QSAR models disappointments: activity cliffs [41]. It is possible because similar molecules can show significantly different biological activities. For these molecules, activities are often mispredicted, even when the overall predictivity of the models are high.

Comparing the activities of the tested molecules it was found that 2-aminobenzimidazole derivatives (compounds **1** – **7**) were more active than 2-methylbenzimidazoles (compounds **8** – **14**). It can be concluded that the presence of an amino substituent leads to an increase in the activity, in comparison to the presence of a methyl group. Also, the analysis of the results indicates that the activity increased in the series of compounds **2** – **3** – **4; 5** – **6** – **7; 9** –**10** – **11** and **12** – **13** – **14**. These observations revealed that the nature of the substituents has an effect on inhibitory activity. It can be concluded that the presence of substituents (CH_3_ or Cl) enhanced the activity of the compounds **2**, **5**, **9** and **12**. However, the presence of a chloro substituent leads to increase in the activity in comparison to the presence of a methyl group. The comparison between antibacterial activity of 1-benzyl and 1-benzoylbenzimidazole derivatives showed that they were similar, except in the cases of compounds **2** *vs*. **5**, and **9** *vs*. **12**, where the presence of carbonyl group leads to decrease in the activity.

## 3. Conclusions

From the results and discussion presented above, we conclude that the 2-amino- and 2- methylbenzimidazole derivatives are effective *in vitro* against the Gram-negative bacteria *Pseudomonas aeruginosa*. The results obtained from present investigation of antibacterial activity studies indicate that the presence of an amino substituent leads to increase in the activity in comparison to the presence of a methyl group. QSAR analysis have been used to study the quantitative effects of the molecular structure of the benzimidazoles on their inhibitory activity. Accurate mathematical models were developed for predicting the antibacterial activity of this class of compounds. The validity of the models have been established by the determination of suitable statistical parameters. From the established QSAR models, it was calculated inhibitory activity of the external set of benzimidazoles and close agreement between experimental and predicted values was obtained. The low residual activity and high cross-validated *r*^2^ values (*r*^2^ _CV_) observed indicated the predictive ability of the developed QSAR models.

## 4. Experimental

### 4.1. Synthesis of Compounds

All the compounds, were synthesized by a general procedure described by Vlaović [42], except for 2-aminobenzimidazole (**1**) and 2-methylbenzimidazole (**8**), which were of commercially available analytical reagent grade.

### 4.2. Antibacterial Investigations

All the benzimidazole derivatives were tested for their *in vitr*o growth inhibitory activity against the Gram-negative bacteria *Pseudomonas aeruginosa* (ATCC 27853). Antibacterial activities of the compounds were tested by the disc-diffusion method under standard conditions using Mueller-Hinton agar medium as described by NCCLS [43]. The investigated isolate of bacteria was seeded in the tubes with nutrient broth. Seeded nutrient broth (1 cm^3^) was taken and homogenized in tubes with melted (45°C) nutrient agar (9 cm^3^). The homogenous suspension was poured in Petri dishes. Filter paper discs (diameter 5 mm) were placed on the cooled medium. The investigated compounds (2·10^−5^dm^3^) were placed by micropipette on formed solid medium and after incubation for 24 hours in a thermostat at 25 – 27°C, inhibition (sterile) zone diameters (including disc) were measured (in mm). An inhibition zone diameter over 8 mm indicates the tested compound is active against microorganism. Every test was done in triplicate.

The MIC measurements were performed by the agar dilution method according to the guidelines established by the NCCLS standard M7-A5 [44]. The MIC of tested benzimidazoles is defined as the lowest concentration of the compound at which no growth of the strain as observed in a period of time and under specified experimental conditions. Stock solutions of the compounds were prepared in DMF. Further dilutions were performed with distilled water. The concentration range of the compounds tested was between 6.25 – 125μg/mL. The inoculated plates were than incubated at 35° C for 16–20h. A control using DMF without any test compound was included for each organisms. There was no inhibitory activity in the wells containing only DMF. The MIC values of the benzimidazoles tested were obtained as μg/mL. In order to classify the antibacterial activity, we established comparisons with antibacterial agents currently employed in therapeutic treatment. The MICs were compared with Ampicillin and Sultamicillin which were screened under similar conditions as reference drugs.

### 4.3. Molecular Modeling

All molecular modeling studies were performed by using HyperChem 7.5 software (HyperCube Inc, Version 7.5) running on a P-III processor [45]. HyperChem includes a model builder that turns a rough 2D sketch of a molecule into a 3D one. The created 3-D models were cleaned up and subjected to energy minimization using MM_2_. The minimization is executed until the RMS gradient value reaches a value smaller than 0.1 kcal/mol · A. The Austin Model-1 method was used for re-optimization until the RMS gradient attains a value smaller than 0.0001 kcal/mol · A using MOPAC. The lowest energy structure was used for each molecule to calculate molecular descriptors.

### 4.4. Descriptor Generation

The numerical descriptors are responsible for encoding important features of the structure of the molecules and can be categorized as electronic, geometric, hydrophobic, and topological characters. Descriptors were calculated for each compound in the data set, using the software HyperChem [45] and Dragon [46]. Since there was a large number of desriptors for each compound, Pearson's correlation matrix was used as a qualitative model, in order to select the suitable descriptors for MLR analysis. The nine descriptors which were showing maximum correlation with inhibitory activity were chosen for further evaluation. The values of descriptors selected for MLR model are presented in Table 3 (molar refractivity (*MR*), polarizability (*P*), molar volume (*MV*), hydration energy (*HE*), molar weight (*MW*), total energy (*TE*), surface area grid (*SAG*), dipole moment (*DM*), and partition coefficient (Clog*P*)).

### 4.5. Statistical Methods

The complete regression analysis were carried out by PASS 2005, GESS 2006, NCSS Statistical Softwares [47]. The ES-SWR algorithm was used to select the most appropriate descriptors. ES-SWR is a popular stepwise technique that combines Forward Selection and Backward Elimination. It is basically a forward selection approach, but at each step it considers the possibility of deleting a variable as in the backward elimination approach, provided that the number of model variables is greater than 2.

## Figures and Tables

**Table 1.** The structures of the compounds studied. 
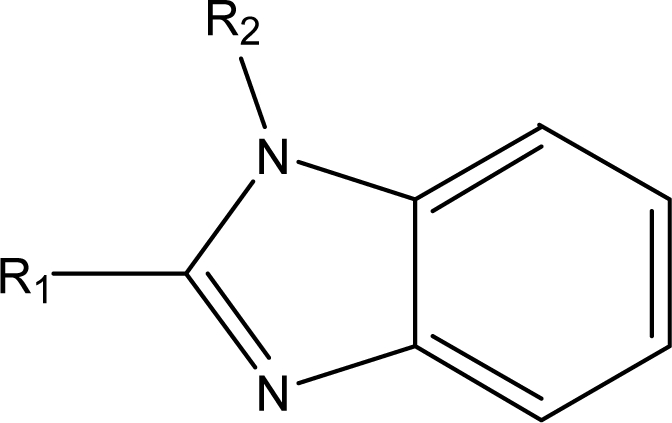

Cmpd	R_1_	R_2_	Cmpd	R_1_	R_2_
**1**	NH_2_	H	**8**	CH_3_	H
**2**	NH_2_	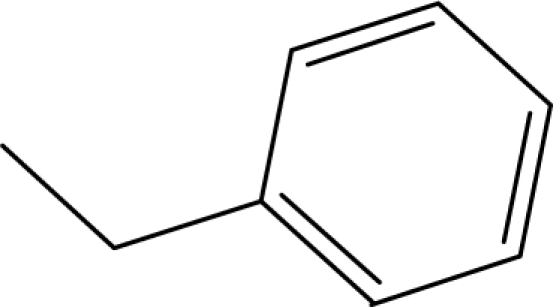	**9**	CH_3_	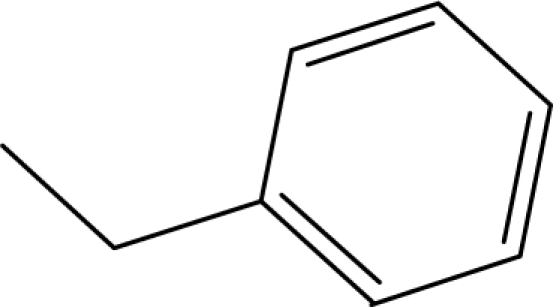
**3**	NH_2_	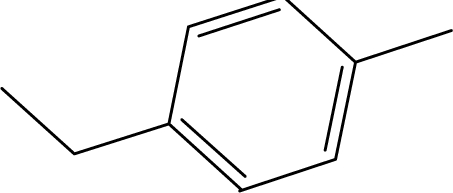	**10**	CH_3_	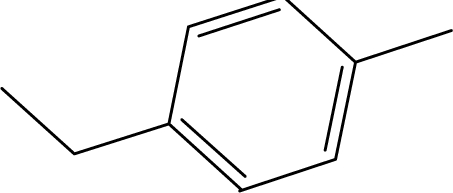
**4**	NH_2_	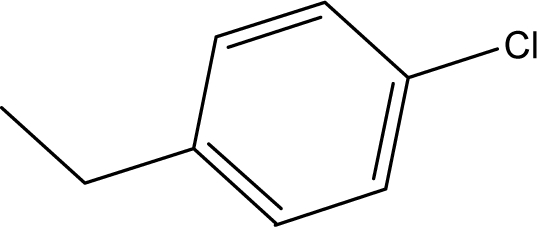	**11**	CH_3_	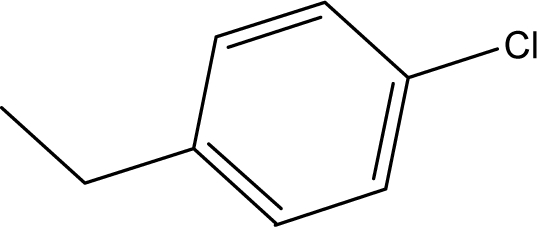
**5**	NH_2_	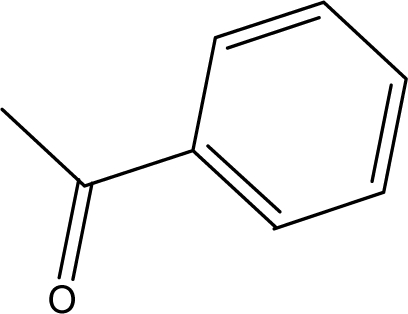	**12**	CH_3_	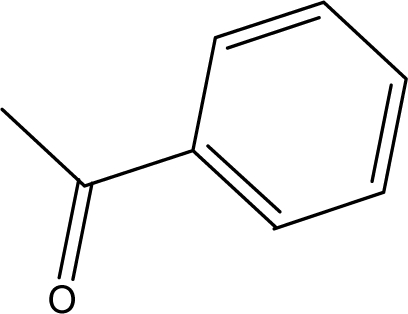
**6**	NH_2_	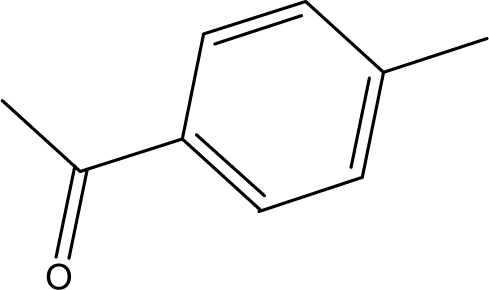	**13**	CH_3_	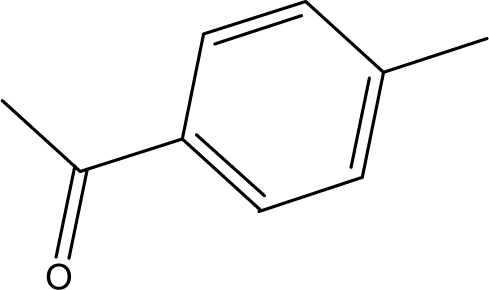
**7**	NH_2_	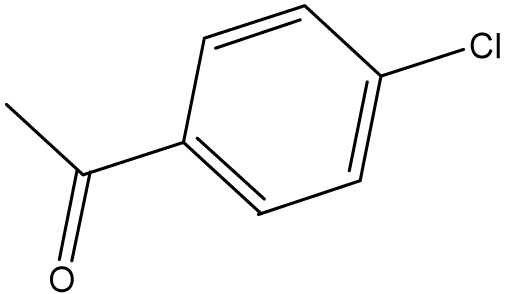	**14**	CH_3_	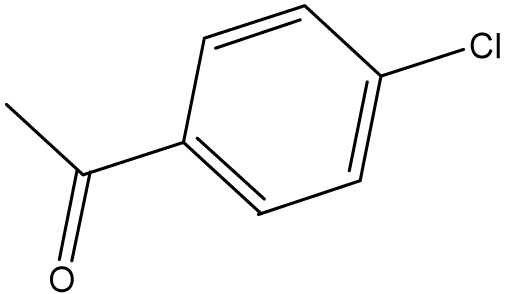

**Table 2.** Antibacterial screening summary.

Compound	MIC (μg/mL)	log1/*c*_MIC_
**1**	50	3.425
**2**	25	3.951
**3**	12.5	4.278
**4**	6.25	4.615
**5**	50	3.676
**6**	12.5	4.303
**7**	6.25	4.638
**8**	100	3.121
**9**	50	3.648
**10**	25	3.975
**11**	12.5	4.312
**12**	100	3.373
**13**	25	4.000
**14**	12.5	4.335
**Ampicillin**	12.5	4.446
**Sultamicillin**	0.78	5.787

**Table 3.** Values of molecular descriptors used in the regression analysis.

Cmpd	*MR*	*P*	*MV*	*HE*	*MW*	*TE*	*SAG*	*DM*	Clog*P*
**1**	43.63	15.13	430.33	−11.28	133.15	−1.21	292.48	1.54	−0.61
**2**	77.28	26.63	675.88	−7.12	223.28	−9.75	416.78	1.45	0.65
**3**	81.56	28.46	728.44	−5.95	237.30	−9.81	442.99	1.53	0.80
**4**	81.99	28.55	712.38	−6.72	257.72	−9.81	437.70	1.48	0.43
**5**	77.21	26.71	666.31	−7.67	237.29	29.80	409.30	2.16	0.07
**6**	81.49	28.55	720.15	−6.52	251.29	29.61	437.71	2.13	0.22
**7**	80.61	28.64	710.59	−7.35	271.71	30.38	434.96	2.88	0.44
**8**	44.83	15.62	450.85	−4.75	132.16	10.35	304.78	1.36	−0.51
**9**	78.48	27.11	693.35	−2.73	222.29	1.34	423.77	1.32	0.75
**10**	82.76	28.94	745.41	−1.61	236.32	1.23	453.96	1.45	0.90
**11**	83.19	29.04	737.17	−2.44	256.73	1.63	448.98	1.69	0.53
**12**	78.41	27.20	686.80	−3.68	236.27	53.89	422.33	2.40	0.17
**13**	82.69	29.03	741.13	−2.53	250.30	53.68	452.01	2.42	0.32
**14**	81.81	29.12	731.39	−3.36	270.71	54.42	447.07	2.86	0.54

**Table 4.** Best MLR models for the prediction of antibacterial activity.

Model	Coefficient		Error	*n*	*r*	*s*	*F*
1	Intercept	2.0228	0.4432	14	0.7910	0.2999	20.053
	*MW*	0.0085	0.0019				
2	Intercept	1.88448	0.3965	14	0.8587	0.2624	15.437
	*MW*	0.0098	0.0018				
	*TE*	−0.0068	0.0031				

**Table 5.** Cross-validation parameters.

Model	PRESS	SSY	PRESS/SSY	S_PRESS_	*r*^2^_CV_	*r*^2^_adj_
1	1.4444	2.8835	0.5009	0.3212	0.4991	0.5944
2	1.2609	2.8835	0.4373	0.3001	0.5627	0.6895

**Table 6.** Predicted log1/c_MIC_ values of benzimidazoles tested against *Pseudomonas aeruginosa.*

Cmpd	log1/c_MIC_exp.	Model 1		Model 2	
		log1/c_MIC_ pred.	Residuals	log1/c_MIC_ pred.	Residuals
**15**	4.328	4.287	0.041	4.298	0.030
**16**	4.278	4.040	0.238	4.030	0.248
**17**	4.314	4.213	0.101	4.233	0.081
**18**	4.333	4.312	0.021	4.337	−0.004
**19**	4.352	4.414	−0.062	4.442	−0.090
